# Illness perception and resilience in patients with cancer: a cross-sectional study

**DOI:** 10.1186/s40359-025-02572-9

**Published:** 2025-03-19

**Authors:** Reza Shabanloei, Mostafa Ghasempour, Reza Zamanesazi, Majid Purabdollah, Mohammad Asghari-Jafarabadi

**Affiliations:** 1 Department of Medical Surgical Nursing, Faculty of Nursing and Midwifery, Tabriz University of Medical Sciences, Tabriz, 51389-47977 Iran; 2 Department of Nursing, Faculty of Nursing, Khoy University of Medical Sciences, Khoy, Iran; 3 Cabrini Research, Cabrini Health, Malvern, VIC 3144 Australia; 4 School of Public Health and Preventive Medicine, Monash University, Melbourne, VIC 3004 Australia; 5 Department of Psychiatry, School of Clinical Sciences, Monash University, Clayton, VIC 3168 Australia

**Keywords:** Illness perception, Resilience, Cancer, Quality of life, Perception

## Abstract

**Background:**

Cancer is the second leading cause of death after cardiovascular diseases and is considered a debilitating and incurable condition. Following diagnosis, individuals often experience anxiety, depression, and diminished social energy. Therefore, identifying factors that influence the psychological state of these patients and intervening to improve their well-being is crucial.

**Aim:**

This study aims to examine the relationship between illness perception and resilience in cancer patients visiting healthcare centers.

**Methods:**

The study was conducted in a cross-sectional design, involving 262 cancer patients selected through stratified random sampling from two public and two private oncology treatment centers in Tabriz, Iran. Data were collected using a demographic checklist, the Connor-Davidson Resilience Scale (CD-RISC), and the Revised Illness Perception Questionnaire (IPQ-R). Data analysis was conducted using IBM SPSS Statistics (V.20) at a significance level of 0.05. Statistical methods included descriptive statistics, one-way ANOVA, t-test, Pearson correlation, and multiple linear regression to examine relationships between demographic variables, illness perception, and resilience.

**Results:**

The majority of participants in the study were male (74%), married (72%), suffering from gastrointestinal cancers (62%), with an average age of 40.9 (SD: 11.9) years. The average overall resilience score was 60.1 (SD: 16.6). Pearson correlation results showed a significant positive correlation between overall resilience and the subscales of illness identity (*r* = 0.26, *p* < 0.001), consequences of illness (*r* = 0.20, *p* < 0.001), personal control (*r* = 0.47, *p* < 0.001), treatment control (*r* = 0.61, *p* < 0.001), and time line cyclical (*r* = 0.33, *p* < 0.001). Linear regression analysis revealed that illness Identity (B = 0.94, CI [0.43, 1.44], *p* < 0.001), personal control (B = 1.75, CI [1.30, 2.21], *p* < 0.001), treatment control (B = 2.37, CI [1.87, 2.88], *p* < 0.001), and time line cyclical (B = 0.30, CI [0.40, 1.01], *p* = 0.04) significantly predicted resilience.

**Conclusion:**

The findings suggest that improving patients’ understanding and control over their illness may enhance their psychological resilience. These results highlight the importance of patient education and psychological interventions in cancer care, aimed at strengthening personal control and resilience. Integrating these strategies into standard care has the potential to improve patients’ ability to cope with the psychological challenges of cancer and ultimately lead to an enhanced quality of life.

## Introduction

Despite significant advancements in medical science, cancer remains one of the most critical diseases of the present century, accounting for approximately 10 million deaths in 2022, making it the second leading cause of death after cardiovascular diseases [1]. According to estimates in 2022, nearly 20 million people were diagnosed with cancer, and the number of new cases is expected to rise to 27 million annually by 2030 [2]. Cancer is the third leading cause of death in Iran, claiming more than 30,000 lives annually. According to the World Health Organization’s projections, cancer incidence in Iran is expected to reach 99,000 cases by 2030, resulting in approximately 62,897 cancer-related deaths. Furthermore, the number of new cancer cases is projected to rise from 112,000 recorded cases in 2016 to nearly 160,000 by 2025, reflecting a 42.6% increase due to elevated cancer risk (13.9%) and demographic changes (28.7%) [3, 4].

Undoubtedly, cancer is one of the most stressful events individuals may face in their lifetime [5]. People diagnosed with this disease can continue to feel its impact for years after the initial diagnosis, as cancer is an extremely unpleasant and often incomprehensible experience [6]. It can lead to a deep existential crisis in daily life, disrupting the patient’s occupation, economic, social status, and family life, while also threatening the future of both the patients and their families [7].

Recent research published in the last few decades emphasizes the detrimental characteristics of life-threatening illnesses such as cancer. Studies have shown that cancer patients often face a wide range of psychological challenges during their illness and even after treatment, including trauma, anxiety, depression, and fear of recurrence. These challenges can significantly impact their emotional well-being and lead to the emergence of maladaptive psychological responses. This highlights the importance of focusing not only on trauma-related outcomes but also on the various psychological issues that cancer patients may experience [8–10]. Psychological theories suggest that resilience is a dynamic and complex process influenced by individual, social, and cultural factors. Not everyone reacts to hardships in the same way, and some individuals are more resilient than others [11, 12]. This variability in resilience can be attributed to various factors such as individual psychological traits, coping strategies, social support, and past experiences with stress or trauma. For example, individuals with stronger emotional regulation, better problem-solving skills, and a sense of purpose tend to show higher resilience. Understanding the elements that shape patients’ responses to their illness, its associated complications, and their resilience against the disease may have significant clinical implications. Such insights can guide the selection of appropriate interventions aimed at supporting cancer patients and survivors in their psychological recovery during the cancer trajectory [11–13].

Resilience helps individuals cope and adapt to difficult and stressful life conditions, protecting them against psychological disorders and life challenges [14]. It is a malleable state within individuals that allows them to maintain, enhance, or restore relatively stable psychological and physical functioning in the face of stressful life events [11, 15]. In other words, resilience can be seen as a dynamic psychological mechanism that explains how individuals cope with unexpected situations. It can be influenced by life conditions, individual environments, situational factors, and contextual backgrounds [11]. In cancer patients, there are numerous and sometimes unexpected pathways to resilience. Resilience pathways in cancer patients involve both direct and indirect processes. Direct pathways include personal traits like optimism, coping skills, and a sense of coherence. Indirect pathways involve psychological adjustments such as benefit-finding, posttraumatic growth, and constructive coping mechanisms. Social support, meaning-making, and environmental stability also facilitate resilience. These factors help patients manage cancer-related stress, promoting emotional well-being and recovery. This comprehensive framework underscores resilience as a dynamic, multifaceted process shaped by individual, social, and contextual influences [12]. Although there is significant variability in how cancer patients confront their illness, there is an increasing recognition today that resilience in the face of life-threatening situations, such as cancer, is much more common than previously thought [12].

Despite reports indicating that higher resilience leads to better coping with cancer [12, 16, 17], there is limited evidence regarding the factors associated with resilience among cancer patients. Studies identifying individual factors related to resilience have highlighted positive characteristics such as the ability to self-regulate or self-control in adverse situations, positive self-perception, autonomy, high self-esteem, problem-solving skills, a strong sense of purpose in life, stress management, and the patients’ understanding of their illness and its aftermath [12, 18].

Therefore, it seems that one of the factors that can affect the resilience of patients diagnosed with cancer is their perception of their illness [19]. Illness perception refers to the organized cognitive and emotional representation an individual has of their disease, its symptoms, and its consequences [20]. Previous studies have emphasized the role of illness perception in the well-being, health, and behaviors of patients diagnosed with chronic and life-altering conditions such as breast cancer and multiple sclerosis [21–23]. According to Albert Bandura’s Social Cognitive Theory, individual’s perception of their illness plays a key role in understanding their health beliefs and health-related behaviors [24, 25]. Another theoretical model that illustrates how illness perception impacts health-related behaviors and outcomes is the Self-Regulation Model proposed by Leventhal and colleagues. Based on the model by Leventhal and colleagues [26], patients regulate their behaviors and emotional responses to illness according to their perceptions of the nature, causes, consequences, controllability, and duration of the disease. Emotional responses such as fear, anxiety, and hope play a significant role in shaping how patients perceive their illness and influence their coping strategies [20]. The model also emphasizes a bidirectional process, where patients’ coping efforts, including resilient coping strategies, can reshape their illness perceptions through continuous feedback. This dynamic interaction ultimately leads to the appraisal stage, where patients assess the effectiveness of their coping strategies and adjust their responses accordingly [26]. Two important aspects of this theory regarding illness perception indicate that, firstly, patients’ beliefs about their conditions often differ from those of their healthcare providers. Secondly, these perceptions can vary significantly among patients, even when they face the same medical conditions [27]. Based on the findings of previous studies, there is evidence to suggest that illness perception can play a significant role in the psychological outcomes of patients and influence their coping behaviors [28–30].

In the past, numerous studies have examined resilience and psychological factors related to cancer, such as emotional regulation, self-efficacy, optimism, coping strategies, social support, stress management, self-esteem, and the ability to find meaning in life [12, 31]. However, only a few have specifically investigated the relationship between illness perception and resilience in cancer patients, particularly within different cultural contexts. Cultural differences can significantly influence resilience in cancer patients by shaping their coping strategies, social support systems, and perceptions of illness. According to a systematic review of cross-cultural measures of resilience, resilience-promoting and protective factors vary across cultures, as beliefs, values, and community support systems can either strengthen or hinder an individual’s ability to cope with the psychological stress of cancer [32]. Furthermore, in the context of cancer survivors in rural areas during the COVID-19 pandemic, factors such as limited healthcare access, family dynamics, and cultural attitudes toward illness and treatment played a critical role in determining the resilience of these individuals [33]. These findings highlight the importance of considering cultural context when designing interventions aimed at enhancing resilience in cancer patients. Also, these studies have identified individual factors such as age and gender, disease-related factors like the presence or severity of physical symptoms, and internal factors such as self-efficacy and hope, as influencing resilience in cancer patients [31]. Studies suggests that illness perception can influence patients’ cognitive appraisal processes, shaping how they interpret their illness and its potential consequences. Patients who perceive their illness as less threatening and hold a more positive view of their condition are likely to adopt more adaptive coping strategies to enhance their resilience. For example, those who see their illness as manageable are more inclined to use problem-focused coping strategies, whereas individuals with more negative perceptions may rely on emotion-focused strategies. Furthermore, patients’ understanding of their illness can impact key components of coping and resilience, such as seeking social support and communicating effectively with healthcare professionals [21, 34, 35]. Although many studies have been conducted on resilience, there is a scarcity of research examining the relationship between illness perception and resilience, and no studies were found on this topic in our country. Additionally, as individual, social, and cultural characteristics can serve as predictors of resilience in patients, there is a need for more evidence from culturally and socially diverse communities to demonstrate the relationship between illness perception and resilience. In line with Leventhal and colleagues’ Self-Regulation Model, sociocultural factors and individual traits play a key role in shaping illness beliefs and perceptions. These factors, including personal experiences, cultural norms, and social support, can significantly influence how patients perceive their illness and cope with the challenges it presents. According to their model, these perceptions, in turn, contribute to resilience, highlighting the need for culturally and socially diverse evidence to better understand the relationship between illness perception and resilience [26]. Therefore, further research is necessary to identify these gaps and provide a deeper understanding of how illness perception might influence resilience in cancer patients, particularly in underexplored cultural contexts such as Iran. Hence, we designed and conducted this study with the aim of investigating illness perception and its relationship with resilience in cancer patients.

## Materials and methods

### Study design

This cross-sectional study was conducted between May and December 2023 among cancer patients visiting two public and two private healthcare centers in Tabriz, the capital of East Azerbaijan Province in northwestern Iran. The study followed the STROBE (Strengthening the Reporting of Observational Studies in Epidemiology) guidelines for conducting and reporting observational research [36].

### Sampling

The sample size (*N* = 260) was determined using G Power with the following parameters: statistical test: linear multiple regression, fixed model, R2 deviation from zero, number of predictors = 6, power = 0.80, α = 0.05, and an effect size of 0.055 based on the squared multiple correlation p2 reported in a study by Xu et al. [37].

Participants were selected using stratified random sampling based on the ratio of visitors to different healthcare centers. The number of patients visiting public healthcare centers was approximately twice that of those attending private centers. Therefore, the sampling was adjusted to maintain this proportion in the selected sample. The inclusion criteria for the study included: a confirmed diagnosis of cancer as determined by a specialist physician, willingness and physical capability to complete the questionnaire, awareness of their condition (verified by asking the patient), and a minimum literacy level to read and write. Questionnaires that were 10% or more incomplete were excluded from the study. Out of 298 distributed questionnaires, after removing distorted or incomplete ones, 262 valid questionnaires remained, resulting in a response rate of 91.87%.

### Survey instrument

In this study, the data collection instrument consisted of three main sections:

1. Demographic Characteristics: This section included questions on age, gender, marital status, education level, duration since diagnosis, type of cancer, average income, and occupation.
2. Connor-Davidson Resilience Scale (CD-RISC): Developed by Conner and Davidson in 2003, this scale comprises 25 items. Based on factor analysis, it is divided into five subscales: personal competence, tolerance of negative effects and resilience against stress, positive acceptance of change, self-control, and spiritual influences. Items are scored on a Likert scale from zero (not true at all) to four (true nearly all the time), with higher average scores indicating greater resilience. Sample items from this standardized questionnaire include statements such as “I can deal with whatever comes my way” (assessing adaptability) and “I tend to bounce back after illness or hardship” (assessing recovery from setbacks). The psychometric properties of scale in Iran by Abdi et al. [38], demonstrating excellent internal consistency with a Cronbach’s alpha of 0.93, indicating high reliability for use in Persian-speaking populations.
3. Revised Illness Perception Questionnaire (IPQ-R): This questionnaire, developed by Moss Morris and colleagues in 2002, includes 38 items covering eight components: illness identity, acute/chronic timeline, consequences of illness, personal control, treatment control, illness coherence, timeline cyclical, and emotional representations. Items are rated on a five-point Likert scale from strongly agree to strongly disagree. Higher scores on the illness identity, consequences of illness, acute/chronic timeline, timeline cyclical, and emotional representations indicate a negative condition, while higher scores on treatment control, personal control, and illness coherence reflect positive beliefs in these areas [39]. Sample items from the standardized IPQ-R questionnaire include “I understand my illness” (measuring illness coherence) and “There is little that can be done to control my illness” (measuring personal control). The Cronbach’s alpha coefficients for the Persian version of this tool were reported as follows: emotional representations (0.93), treatment control (0.85), consequences of illness (0.78), acute/chronic timeline (0.84), coherence (0.86), personal control (0.78), timeline cyclical (0.38), psychological traits (0.75), immune system factors (0.75), and risk factors (0.23) [40].

To utilize this tool, the face and content validity of the Persian version was assessed by 11 faculty members from Tabriz University of Medical Sciences, and minor adjustments were made based on their feedback. Based on the feedback received, minor adjustments were made to certain questionnaire items to enhance clarity, comprehension, and cultural relevance. For example, some illustrative examples were added in parentheses to specific items. These revisions were reviewed and approved by the research team prior to data collection. Additionally, the internal consistency reliability of the questionnaire was calculated in a pilot study with 30 cancer survivors attending selected centers (from both public and private health centers). The results showed that the Cronbach’s alpha coefficient was 0.87 for the Connor-Davidson Resilience Scale (CD-RISC) and 0.89 for the Moss Morris Illness Perception Questionnaire.

### Ethical considerations

After obtaining permission from the Research Council and ethical approval from the Regional Research Ethics Committee of Tabriz University of Medical Sciences (IR.TBZMED.REC.1398.899), the researcher visited the selected centers in Tabriz. With the approval of the respective center management, data collection was initiated. After identifying the patients who met the criteria for entering the study, the researcher communicated with them and explained the objectives of the research to them; After accepting the patient’s participation, informed consent was obtained from all of the patients and questionnaires were provided to them and they were requested to complete the mentioned questionnaires.

### Statistical methods

IBM SPSS Statistics (V.20) (IBM SPSS Statistics, IBM) software was used for data analysis at the alpha level of 0.05. Sociodemographic characteristics were described using frequencies (percentages) for categorical variables and means (standard deviations) for numerical variables. One-way ANOVA was conducted to compare the mean resilience scores across different demographic groups, while independent samples t-tests were used where comparisons involved two groups. Pearson correlation coefficients assessed the relationship between illness perception and resilience scores. Multiple linear regression analyses were performed to evaluate the impact of illness perception and other demographic variables on resilience. Before linear multiple regression analysis, categorical variables were coded into dummy variables. The skewness (within±1.5) and kurtosis (within±2) showed that the data follow a normal distribution. The assumptions of linear regression including the residual independence, residuals normality, residuals homoscedasticity, and linearity of the relationship between the variables, as well as the collinearity using variance inflation factor (VIF) were checked and confirmed.

## Results

### Participants

In this study, 262 cancer patients visiting the selected treatment center participated, with a mean age of 40.87 years (SD: 10.73). The majority of participants were male (74%), married (72%), and diagnosed with gastrointestinal cancers (62%). Approximately 73% of them had been diagnosed with cancer within the past 1 to 12 months, 50% of study participants had a history of hospitalization at least 1 to 3 times after being diagnosed with cancer. Further details regarding the demographic information of the participants are provided in Table 1.

**Table 1:** Sociodemographic characteristics and their relationship with resilience in patients with cancer (*N* = 262)

Demographic Characteristics	Grouping	frequency	Percentage	*P*-value (Significance Level)
Gender	Male	194	74	0.27
	Female	68	26	
Marital Status	Single	73	27.9	0.61
	Married	89	72.1	
Education Level	Below Diploma	86	32.8	*P* < 0.001
	Diploma	80	30.5	
	Bachelor	90	34.4	
	Higher	6	2.3	
Employment Status	Student	7	2.7	*P* < 0.001
	Unemployed	63	24	
	Self-Employed Job	86	32.8	
	Housewife	38	14.5	
	Government’s Employee	49	18.6	
	Retired	19	7.3	
Living situation	single	12	4.6	0.33
	with parents	68	26	
	with spouse	54	20.6	
	with spouse and children	128	48.9	
Economic situation	incomes < expenses	153	58.4	0.05
	incomes = expenses	73	27.9	
	incomes ˃ expenses	36	13.7	
Type of Cancer	Head And Neck	46	17.6	0.02
	Gastro-Intestinal System	163	62.2	
	Urinary-Genital System	24	9.2	
	Thorax (Breast, Lung, Etc.)	29	11.1	
Type of Treatment	Chemotherapy	243	92.7	0.07
	Surgery	13	5	
	Radiotherapy	6	2.3	
Awareness Duration of Illness	1–12 month	191	72.9	0.27
	13–24 month	54	20.6	
	More than 2 years	17	6.5	

*P*-value were computed using t-test or Analysis of variance (ANOVA)

In this study, the mean total resilience score of participating patients was 59.52 (SD: 16.64). Based on normalized scores, the highest and lowest scores were related to the dimensions of spiritual impacts and self-control, respectively (See Table 2). Additionally, the results of one-way ANOVA tests indicated that the mean total resilience score significantly differed based on patients’ educational levels (*P* <0.001), economic status (*P* = 0.05), employment type (*P* <0.001), and the type of cancer (*P* = 0.02) they were diagnosed with. Post hoc tests using Tukey HSD revealed that the average resilience among patients with a bachelor’s degree (*P* <0.001), retirees (*P* <0.001), those with Urogenital cancers (*P* <0.001), and those whose income matched their expenses ( *P* = 0.02) was significantly higher than that of other compared groups.

**Table 2:** Average total score of resilience, disease perception and its dimensions in cancer patients participating in the present study

	Variable	Acquirable domain	Mean (SD)
Resilience	Personal competence, high standards, and tenacity	0–32	9.21(6.23)
	Trust in one’s instincts, tolerance of negative affect, and strengthening effects of stress	0–28	17.03(4.91)
	Positive acceptance of change and secure relationships	0–20	12.17(4.29)
	Control	0–12	6.80(2.41)
	Spiritual influences	0–8	4.90(1.8)
	Total score	0–100	59.52(16.64)
Illness Perception	illness identity	1–14	8.12(2.96)
	Timeline (Acute/Chronic)	6–30	17.41(3.24)
	Consequences of illness	6–30	20.4(4.24)
	Personal Control	6–30	18.72(3.29)
	Treatment control	5–25	16.51(3.30)
	Illness Coherence	5–25	13.53(3.62)
	Time line cyclical	4–20	11.85(2.4)
	Emotional Representations	6–30	19.22(4.20)

The findings of this study indicated that after normalizing the scores of individual subscales to a 0–5 range for comparability, the highest and lowest scores were associated with the components of treatment control (3.3 out of 5) and illness coherence (2.75 out of 5), respectively (see Table 2). Additionally, Pearson correlation coefficient results demonstrated significant positive correlations between the total resilience score and the components of disease nature (*r* = 0.26, *p* < 0.001), consequences of illness (*r* = 0.20, *p* < 0.001), personal control (*r* = 0.47, *p* < 0.001), treatment control (*r* = 0.61, *p* < 0.001), and time line cyclical (*r* = 0.33, *p* < 0.01). However, no significant relationship was found between the total resilience score and the components of acute/chronic timeline, illness coherence, and emotional representations (*p* > 0.05) (See Table 3).

**Table 3:** Correlation between the components of illness perception and the total score of resilience and its components in participating cancer patients

	Total Resiliency	illness identity r (*p*-value)	Time line acute chronic r (*p*-value)	Consequences of illness r (*p*-value)	Personal Control r (*p*-value)	Treatment control r (*p*-value)	Illness Coherence r (*p*-value)	Time line cyclical r (*p*-value)	Emotional representations r (*p*-value)
Total Resiliency	1	0.26(0.001) **	0.06(0.35)	0.20(0.001) **	0.47(0.001) **	0.61(0.001) **	.02(0.77)	0.33(0.001) **	0.00(1.00)
Illness identity		1	-0.01(0.90)	0.98(0.12)	-0.09(0.90)	0.96(0.12)	-0.04(0.48)	0.17(0.005)	-0.028(0.65)
Time line acute chronic			1	0.16(0.01) *	0.32(0 < 0.001) **	0.13(0.03)	0.26(0 < 0.001) **	0.14(0.02) *	0.03(0.61)
Consequences of illness				1	0.20(0.002) **	0.29(0 < 0.001)	0.12(0.06)	0.38(0 < 0.001) **	0.42(0 < 0.001) **
Personal Control					1	0.33(< 0.001)	0.18(0.004) **	0.18(0.004) **	0.13(0.03) *
Treatment control						1	-0.07(0.24)	0.29(0 < 0.001) **	0.01(0.83)
Illness Coherence							1	0.35(0 < 0.001) **	0.28(0 < 0.001) **
Time line cyclical								1	0.41(0 < 0.001) **
Emotional representations									1

^**^Correlation is significant at the 0.01 level (2-tailed)

^*^Correlation is significant at the 0.05 level (2-tailed)

For examine the impact of illness perception variables and demographic factors on resilience, a multiple regression analysis using the Enter method was conducted. In this analysis, resilience was treated as the dependent variable, while the components of illness perception and demographic variables were entered as independent variables. The results of the multiple regression indicated that the overall model significantly predicted variations in resilience (R^2^ = 0.72, F = 21.32, *p* < 0.001). The regression analysis results (Table 4) revealed significant positive relationships between resilience and several components of illness perception before and after adjusting for variables. However, after adjusting for these variables, only the components of illness Identity (B = 0.94, CI [0.43, 1.44], *p* < 0.001), personal control (B = 1.75, CI [1.30, 2.21], *p* < 0.001), treatment control (B = 2.37, CI [1.87, 2.88], *p* < 0.001), and time line cyclical (B = 0.30, CI [0.40, 1.01], *p* = 0.04), and some demographic variables such as age (B = 0. 21, CI [0.52, 0.38], *p* = 0.01), living situation (B = 5.74, CI [0.90, 10.57], *p* = 0.02), economic status (B = -4.765, CI [-8.00, -1.52], *p* = 0.004), and the number of chemotherapy sessions (B = 1.29, CI [0.90, 1.63], *p* < 0.001) showed significant correlations with resilience (see Table 4).

**Table 4:** The results of linear regression analysis of resilience based on the components of disease perception and demographic variables

Covariates	Unadjusted estimates				Adjusted estimates			
	**B**	**95% CI**		***p* value**	**B**	**95% CI**		***p* value**
		L	U			L	U	
Age	-.025	-.21	.16	.79	.215	.052	.378	.01
Sex								
Male	2.95	-1.66	7.57	0.21	3.55	-.17	7.28	.06
Female	Referent	-	-	-	-	-	-	-
Marital Status								
Single	3.67	-.82	8.18	0.11	3.03	-.55	6.61	.09
Married	Referent	-	-	-	-	-	-	-
Education Level								
Below Diploma	-1.453	-14.706	11.799	.829	-8.603	-18.50	1.29	.088
Diploma	8.775	-4.510	22.060	.195	-1.439	-11.25	8.37	.773
Bachelor	9.678	-3.556	22.911	.151	.900	-8.92	10.72	.857
Higher	Referent	-	-	-	-	-	-	-
Employment Status								
Student	-3.669	-18.139	10.801	.618	6.538	-3.747	16.823	.212
Unemployed	-8.479	-17.044	.087	.052	-3.456	-9.233	2.321	.240
Self-Employed Job	-7.073	-15.369	1.223	.094	.903	-4.839	6.645	.757
Housewife	-5.158	-14.353	4.038	.270	2.189	-4.684	9.061	.531
Government’s Employee	-9.588	-18.432	-.743	.034	-3.248	-9.905	3.409	.337
Retired	Referent	-	-	-	-	-	-	-
Living situation								
single	-3.711	-13.512	6.090	.45	5.651	-1.355	12.656	.113
with parents	.568	-4.303	5.440	.81	5.739	.904	10.574	.020
with spouse	-6.850	-12.118	-1.582	.01	-4.664	-8.209	-1.119	.010
With spouse and children	Referent		-	-	-	-	-	-
Economic situation								
incomes < expenses	-6.14	-10.752	-1.531	.009	-4.765	-8.005	-1.525	.004
incomes ˃ expenses	-7.15	-13.752	-.551	.034	-7.355	-11.957	-2.752	.002
incomes = expenses	Referent	-	-	-	-	-	-	-
Type of Cancer								
Head And Neck	2.600	-5.113	10.312	.507	4.135	-.637	8.906	.089
Gastro-Intestinal System	-2.291	-8.846	4.265	.492	.067	-3.945	4.080	.974
Urinary-Genital System	5.784	-3.191	14.760	.206	5.547	-.127	11.220	.055
Thorax (Breast, Lung, Etc.)	Referent	-	-	-	-	-	-	-
Type of Treatment								
Chemotherapy	-6.272	-19.800	7.257	.362	-4.651	-14.372	5.070	.347
surgery	.205	-15.952	16.362	.980	-8.892	-20.326	2.541	.127
Radiotherapy	Referent	-	-	-	-	-	-	-
The number of times chemotherapy	.74	.140	1.350	.016	1.289	.896	1.683	< 0.001
Illness Identity	1.474	.813	2.135	< 0.001	.940	.437	1.443	< 0.001
Consequences of illness	.841	.347	1.336	.001	-.257	-.638	.124	.185
Personal Control	2.40	1.861	2.947	< 0.001	1.756	1.297	2.215	< 0.001
Treatment control	3.087	2.601	3.573	< 0.001	2.378	1.875	2.881	< 0.001
Time Line Acute Chronic	0.3	-0.32	0.92	0.35	-0.67	-1.147	0.196	0.06
Time line cyclical	2.34	1.514	3.164	< 0.001	0.304	0.405	1.013	0.04

F = 21.32, *p* < 0.001, adjust R2 = 0.72

## Discussion

The present study aimed to determine illness perception and its relationship with resilience in cancer patients visiting healthcare centers in Tabriz. The findings revealed that cancer patients reported moderate levels of resilience and illness perception. Furthermore, the study showed that as patients’ understanding and control over their illness increased, so did their resilience. The key contribution of this study lies in its detailed examination of this relationship within the unique cultural context of Iran, which has been underexplored in previous research. More specifically, this research highlights how certain individual and social characteristics, as well as dynamic components of illness perception, such as personal and treatment control, can significantly predict resilience levels. These insights provide a foundation for developing targeted interventions aimed at improving illness perception to enhance psychological resilience. Improving patients’ understanding of their illness can help reduce anxiety and fear associated with cancer diagnosis and treatments [20]. These interventions may include educational programs aimed at enhancing illness coherence, promoting realistic beliefs about the controllability of the illness, and increasing patients’ sense of personal control. Additionally, cognitive-behavioral therapy (CBT) and psychoeducation could be integrated into these interventions to help patients develop more adaptive coping strategies and enhance their psychological resilience [41]. By addressing illness perception in these ways, we can not only improve patients’ emotional well-being but also enhance their ability to cope effectively with the challenges posed by cancer and its treatments [21–23]. Therefore, this study not only fills a critical gap in the literature by addressing the cultural and psychological factors influencing resilience in a non-Western population, but it also opens new avenues for future research and practical applications in cancer care.

The findings of the present study indicated that the average resilience score among cancer patients participating in the study was within the moderate range. In recent years, several studies have been conducted in the Iranian context to examine the resilience of cancer patients. The results of these studies align with the findings of the current study, reporting similar moderate resilience scores among cancer patients [42–44]. While these studies have primarily focused on reporting resilience levels, they have not explored specific coping mechanisms used by patients. This highlights the need for future research examining how cancer patients develop and apply coping strategies to manage the psychological challenges associated with their illness, emphasizing that despite these challenges, many patients have developed some coping mechanisms but still require additional psychological and social support. Examples of coping mechanisms identified in the literature include active coping, minimizing the situation, avoidance coping, positive reappraisal, seeking social support, problem-focused coping, religious coping, reliance on God, distraction, acquiring information and education, and maintaining a positive attitude [12, 45, 46].

In line with the present study, research by Gao et al. [47] in China and Fradelos et al. [48] in Greece reported moderate resilience levels in cancer patients. Similarly, Zahid et al. [49] in Pakistan found higher resilience scores in head and neck cancer patients, which may be attributed to the use of the Resilience Scale-14 (RS-14) in their study. In Sweden, a study in 2020 found higher resilience scores in newly diagnosed breast cancer patients using the CD-RISC-25 [50]. On the other hand, a study from China reported lower resilience scores in breast cancer patients compared to our study [51]. These differences could be attributed to cultural variations, the type of cancer, the healthcare services provided in different countries, and the stages of disease progression. Therefore, comparing these results with other studies is significant, as they highlight the cultural, social, and psychological differences that affect resilience. Such comparisons contribute to a better understanding of the factors influencing resilience in different populations and aid in developing intervention strategies tailored to the specific needs of each group.

Furthermore, statistical analyses revealed that factors such as education level, economic status, employment type, and cancer type significantly influenced resilience. Studies by Festerling et al. [52], and Hu et al. [53] also confirmed the correlation between socio-economic factors such as economic status, education level, and employment conditions and resilience. Kordan et al. [54] and Wu et al. [55] found that higher education levels were associated with greater resilience, consistent with the findings of this study. In contrast, Abdollahzadeh et al. [56] in Iran found significant gender-based differences in resilience, while no such relationships were observed in our study. These discrepancies could arise from differences in demographic characteristics, cancer type, or assessment methods used. Similarly, the study by Gao et al. [47] found no significant correlation between resilience scores and demographic variables such as age, gender, body mass index, occupation, income, type of treatment, or family history of cancer. However, resilience showed significant differences based on marital status, education level, and metastatic status. The discrepancies between our study and other research may stem from differences in the demographic characteristics of participants, cultural variations, or the type and stage of cancer. Furthermore, differences in resilience assessment methods and statistical tools, such as regression models, ANOVA, t-tests, or Structural Equation Modeling (SEM), used in various studies may also account for these variations.

In the current study, the mean normalized scores of the various components of illness perception were within a moderate range. The lowest and highest scores among these components were related to “illness coherence” and “treatment control,” respectively. These results suggest that while patients feel they have greater control over their treatment process, they may have a less clear understanding of the nature and causes of their illness. This highlights the importance of medical information and suggests a need to enhance patient education regarding the nature of their disease.

In this questionnaire, high scores on the components of illness identity (symptoms), illness consequences, acute/chronic timeline, timeline cyclical, and emotional representations indicate a negative situation for the individual on these scales. However, high scores on treatment control, personal control, and illness coherence suggest positive beliefs in these areas [39]. Supporting these findings, a study by Saritas et al. in Turkey [57], using the IPQ-R questionnaire with 318 cancer patients (of various types), reported results very similar to the current study. Similarly, another study conducted in Turkey in 2019, aimed at assessing illness perception in 380 women with breast cancer using the IPQ-R, showed that the mean scores of illness perception components were quite close to those in the current study, with the main difference being that in the mentioned study, the highest score was related to the personal control component [34]. Additionally, in a study by Shabahang et al. [58], the mean scores for various components (identity component, timeline component, treatment control, illness coherence, serious consequences, personal control and.

emotional representations) were also similar to the results of the current study, with personal control having the highest score in that research as well. The difference in findings can be attributed to the fact that unlike the current study, the participants in the last two studies were exclusively breast cancer patients. It is worth noting that although the incidence rate of breast cancer has increased over the past four decades, advancements in early detection and the development of effective treatment protocols, such as targeted therapies and hormone-based treatments, have led to a steady decline in breast cancer mortality since its peak in 1989 [59]. As a result, patients may perceive a greater sense of personal control over their health outcomes. Furthermore, the rise of public awareness campaigns and support systems specifically designed for breast cancer patients may further enhance their sense of empowerment and personal agency, ultimately influencing their illness perception more positively compared to patients with other types of cancer.

The results also revealed a significant positive correlation between overall resilience scores and the components of illness identity, illness consequences, personal control, treatment control, and time line cyclical. These components can serve as predictors of resilience in cancer patients. Similarly, Milinavičienė et al. [60] demonstrated that at the end of oncogynecological rehabilitation, higher resilience in women was correlated with higher personal control, better treatment control, better illness coherence, lower emotional representations, fewer illness consequences, and a lower timeline cyclical. In line with the present study, other research conducted among hemodialysis patients [61], diabetic patients [62], and individuals with psoriasis [63] has also shown significant positive correlations between resilience and illness perception. These findings, consistent with the current study, highlight the importance of interventions aimed at improving patients’ understanding of their illness and enhancing their sense of personal and treatment control. The positive and significant correlations between overall resilience and components suggest that patients with a better understanding of these aspects demonstrate higher levels of resilience. Specifically, the strong correlation between resilience and both treatment control and personal control emphasizes the importance of a sense of control and an active role in managing one’s treatment. On the other hand, the absence of a significant correlation between resilience and components such as illness coherence and emotional representations may indicate that patients’ understanding of the long-term aspects of the disease or their emotional reactions does not significantly influence their resilience. This finding suggests that cognitive and behavioral factors might play a more prominent role in cancer patients’ resilience compared to emotional factors. While some research indicates that emotional representations, including patients’ perceptions of illness-related emotions such as fear, hope, anxiety, and distress, can influence resilience, other studies emphasize the critical role of cognitive and behavioral coping strategies [64, 65]. These studies suggest that interventions such as cognitive reframing, problem-solving skills, and goal-setting behaviors may, in certain contexts, exert a stronger and more significant impact on resilience compared to emotional responses alone [66, 67]. These findings align with the results of the present study, suggesting that resilience in cancer patients may also be driven by cognitive and behavioral processes. These results underscore the need for multidimensional therapeutic approaches that, beyond managing the physical aspects of the disease, also focus on enhancing patients’ understanding of their illness and treatment.

### Limitations and strengths

This study had several limitations. First, the generalizability of the study is a concern; since the sampling of cancer patients was conducted from a specific geographical area, the results may not be applicable to other geographical or cultural contexts. Second, the study design chosen was cross-sectional; using this design limits the ability to examine causal relationships between variables, and longitudinal research may provide more precise insights into the relationships among variables. Third, the type of instrument used is another limitation; the use of self-report tools may be subject to response bias, which can affect the accuracy and honesty of respondents’ answers. Fourth, there were some confounding variables that were not controlled for in this study, which could influence resilience and illness perception. In this study, multiple linear regression analyses were conducted to control for the impact of confounding variables on resilience and illness perception. The results of these analyses are reported in the Results section. However, given the complex nature of psychological constructs such as resilience and illness perception, certain unmeasured confounders may have influenced the findings. Future research could address this limitation by incorporating additional control variables and employing more comprehensive models to ensure a deeper understanding of these constructs. However, this study also had significant strengths. First, it provided a comprehensive examination of various components of illness perception and demographic variables and their impact on the resilience of cancer patients, which is a notable strength. Second, the use of a large sample of cancer patients increased the statistical power of the study, yielding reliable results. Third, the use of multiple regression analyses with the Enter method helped identify significant and influential variables affecting resilience. Fourth, the findings of this study could contribute to developing effective intervention programs to enhance the resilience of cancer patients and improve their quality of life. Overall, this study, by providing new insights into the effects of illness perception components and demographic variables on the resilience of cancer patients, can aid in developing more optimized strategies to support these patients. However, future research could achieve greater accuracy and validity by better controlling for confounding variables and utilizing longitudinal designs.

## Conclusion

The results of the current study indicate that the cancer patients participating in this research exhibited a moderate level of resilience and illness perception. The findings suggest that improving patients’ understanding and control over their illness may enhance their psychological resilience. These results highlight the importance of patient education and psychological interventions in cancer care, aimed at strengthening personal control and resilience. Therefore, identifying and integrating appropriate interventions into standard care, with the goal of improving patients’ illness perception, has the potential to enhance their ability to cope with the psychological challenges of cancer and ultimately improve their quality of life.

## Data Availability

The data that support the findings of this study are available from the corresponding author upon reasonable request.

## Abbreviations

CD-RISC: Connor-Davidson Resilience Scale
IPQ-R: Revised Illness Perception Questionnaire
SD: Standard deviation
CI: Confidence interval
COVID-19: Coronavirus disease of 2019
STROBE: Strengthening the Reporting of Observational Studies in Epidemiology
One-way ANOVA: One-way analysis of variance
VIF: Variance inflation factor
R^2^: R-squared
CBT: Cognitive-Behavioral Therapy
RS-14: Resilience Scale-14
SEM: Structural Equation Modeling
